# Targeting of PBP1 by β-lactams Determines *recA*/SOS Response Activation in Heterogeneous MRSA Clinical Strains

**DOI:** 10.1371/journal.pone.0061083

**Published:** 2013-04-23

**Authors:** Konrad B. Plata, Sarah Riosa, Christopher R. Singh, Roberto R. Rosato, Adriana E. Rosato

**Affiliations:** 1 Department of Pathology and Genomic Medicine, The Methodist Hospital, Houston, Texas, United States of America; 2 Center for Molecular and Translational Human Infectious Diseases Research, The Methodist Hospital Research Institute, Houston, Texas, United States of America; University of Edinburgh, United Kingdom

## Abstract

The SOS response, a conserved regulatory network in bacteria that is induced in response to DNA damage, has been shown to be associated with the emergence of resistance to antibiotics. Previously, we demonstrated that heterogeneous (HeR) MRSA strains, when exposed to sub-inhibitory concentrations of oxacillin, were able to express a homogeneous high level of resistance (HoR). Moreover, we showed that oxacillin appeared to be the triggering factor of a β-lactam-mediated SOS response through *lexA/recA* regulators, responsible for an increased mutation rate and selection of a HoR derivative. In this work, we demonstrated, by selectively exposing to β-lactam and non-β-lactam cell wall inhibitors, that PBP1 plays a critical role in SOS-mediated *recA* activation and HeR-HoR selection. Functional analysis of PBP1 using an inducible PBP1-specific antisense construct showed that PBP1 depletion abolished both β-lactam-induced *recA* expression/activation and increased mutation rates during HeR/HoR selection. Furthermore, based on the observation that HeR/HoR selection is accompanied by compensatory increases in the expression of PBP1,-2, -2a, and -4, our study provides evidence that a combination of agents simultaneously targeting PBP1 and either PBP2 or PBP2a showed both *in-vitro* and *in-vivo* efficacy, thereby representing a therapeutic option for the treatment of highly resistant HoR-MRSA strains. The information gathered from these studies contributes to our understanding of β-lactam-mediated HeR/HoR selection and provides new insights, based on β-lactam synergistic combinations, that mitigate drug resistance for the treatment of MRSA infections.

## Introduction

*Staphylococcus aureus* is a main pathogen responsible for a number of diseases ranging from skin and soft tissue infections to life-threatening endocarditis, both in hospitals and community settings [1]. In *S. aureus*, the cell wall is a large mesh-like polymer where the main constituent, the peptidoglycan polymer, is made of long glycan chains cross-linked with peptide bridges [2]. The primary target of β-lactam antibiotics are penicillin binding proteins (PBPs), which are involved in the last stages of peptidoglycan biosynthesis [2]. β-lactam resistance in methicillin-resistant *S. aureus* (MRSA) involves the acquisition of PBP2a, a protein encoded by *mecA*, to complement the four native staphylococcal PBPs (PBP1-4). In MRSA strains, native PBP2 has been shown to be required for expression of resistance, while in methicillin-susceptible *S. aureus* (MSSA) strains it is essential for growth [3], [4]. PBP1 localizes at the division septum which is the main site of cell wall synthesis in *S. aureus*. It has also been shown to be critical for growth and cell division and separation, but not the major contributor of peptidoglycan cross-linking in both MRSA and MSSA [5]–[7]. Orthologs of *S. aureus* PBP1 are PBP3 in *Escherichia coli* and *Caulobacter crescentus* and PBP2B in *Bacillus subtilis*; in all cases, they have been shown to be involved in peptidoglycan synthesis during cell division [8]–[10]. Both PBP1 and PBP2 are the essential PBPs in *S. aureus*, but only PBP1 remains active in the presence of PBP2a, which can replace the essential transpeptidase function of PBP2 [11]. PBP4, although not essential for viability, has been shown to play a key role in β-lactam resistance in community-acquired MRSA (CA-MRSA), an effect that is linked to its unique function in producing highly-cross linked peptidoglycan [12].

A feature of MRSA strains from both hospital- and community-associated infections is their heterogeneous expression of resistance to β-lactam antibiotics [13]–[15], in which the majority of the cells express resistance to low concentrations of oxacillin [i.e., ≥10 µg/ml; heterotypic resistance (HeR)], while the minority of cells (≤0.1%) expresses resistance to a high concentration [i.e., ≥256 µg/ml; homotypic resistance (HoR)] [13]–[16].

In previous studies, we demonstrated that MRSA-HeR strains, clinically misinterpreted as MSSA (MICs to oxacillin: 2 µg/ml), were able to express a homogeneous, high level of resistance (MICs: 256 µg/ml; HoR) when exposed to sub-inhibitory concentrations of oxacillin (0.5 µg/ml). MRSA-HoR strains are stable and retain their trait of high level of resistance after several passages in drug-free media. We determined the pre-existence of a hypermutable sub-population in MRSA-HeR strains that favored the selection to HoR phenotype in the presence of oxacillin [15]. Oxacillin appeared as the triggering factor of a β-lactam-induced SOS response through *lexA/recA* regulators, responsible for an increased mutation rate and selection of the highly resistant HoR derivative [15]. The activated LexA/RecA complex induces autocleavage of the repressor LexA leading to the transcription of genes involved in DNA repair. Moreover, an error-prone polymerase (*umuC*) has been identified within the *S. aureus lexA* regulon as being involved in the mutation rate [17].

Previous works have shown that: 1)- β-lactam antibiotics that target the transpeptidase domain of PBP3 (ceftazidime) and do not directly damage DNA or affect replication in *E.coli*, trigger the SOS response *via* the two-component system DpiAB [18]; and 2)- inhibition of cell wall biosynthesis at steps other than PBP3 activity may specifically induce DNA Pol IV expression in *E.coli*, contributing to an increased mutation rate [19]. Based on these and our own observations that β-lactam-mediated SA13011 HeR/HoR selection involves induction of the SOS response, we hypothesized that the β-lactams-induced SOS response in heterogeneous MRSA may originate under circumstances in which cell wall integrity is compromised. The purpose of the following studies was to test this hypothesis, and to elucidate whether signals from cell wall components are involved in triggering *recA*-mediated SOS response in SA13011-HeR, allowing for HoR resistant phenotype selection and survival in the presence of β-lactams. In this context, by selectively exposing MRSA SA13011-HeR cells to β-lactams and non-β-lactam cell wall inhibitors, we determined that PBP1 plays a critical role in *recA* activation and SOS-mediated HeR/HoR selection. Functional analysis of PBP1 with an inducible PBP1-specific antisense RNA demonstrated that PBP1 depletion may lead to decreased *recA* expression during HeR/HoR selection, causing a decrease of mutation rate through *UmuC*. Furthermore, based in the mechanistic observations established for PBP1, our study provides evidence that combination of agents targeting PBP1 and β-lactams targeting PBP2 and/or PBP2a showed *in-vitro* as well *in-vivo* efficacy, representing a therapeutic option for the treatment of highly-resistant MRSA-HoR. Our results provide an important contribution to our understanding of β-lactam-mediated HeR/HoR selection and new insights for the treatment of MRSA infections.

## Materials and Methods

### Strains, growth conditions and antibiotics used in this study

All of the strains and plasmids used in this study are listed in Table 1. Antibiotics oxacillin (OXA), cloxacilin (CLOX), ceftobiprole (BAL), cefotaxime (CTX), cefoxitin (FOX), cefaclor (CEC), imipenem (IMP), bacitracin (BAC), D-cycloserine (DCS) and vancomycin (VAN) were obtained from Sigma-Aldrich (St. Louis, MO). Antimicrobial susceptibility tests were determined according to the guidelines of the Clinical and Laboratory Standards Institute [20]. Trypticase soy agar with 5% sheep blood (Becton, Dickinson and Company, Sparks, MD), Mueller-Hinton (MH) agar (BBL Microbiology Systems, Cockeysville, MD), Trypticase Soy Agar (BBL Microbiology System, Cockeysville, MD), LB broth (Difco, BD Biosciences), supplemented with appropriate antibiotics when necessary (Sigma, St. Louis, MO; US Biochemicals, Cleveland, OH) were used for subculture and maintenance of *S. aureus* strains. *E.coli* was grown and maintaned in Difco LB broth and Difco LB agar.

**Table 1:** Strains, plasmids, and primers used in this study.

Strain or plasmid	Description	Reference
SA13011-HeR	Heterogeneous-*mecA*(+) oxacillin susceptible	[15]
SA13011-HoR	SA13011-HeR+OXA (0.5 µg/ml); SA13011 homogeneous derivative	[15]
LMR-21	SA13011-HeR *recA*-lacZ	This study
LMR-22	LMR-21+OXA (0.5 µg/ml); LMR-21 homogeneous derivative	This study
LMR-23	SA13011-HeR+pCL15-*pbp1*T-ANT	This study
LMR-24	LMR23+OXA (0.5 µg/ml); LMR-23 homogeneous derivative	This study
LMR-25	SA13011-HeR+pCL15-*pbp1*T-EV (empty-vector)	This study
LMR-26	LMR-25+OXA (0.5 µg/ml); LMR-25 homogeneous derivative	This study
**plasmids**		
pMC1871		[26]
pRB473		[55]
pCL15		[28]
pMC1871-*recA lacZ*		This study
pRB473-*recA lacZ*		This study
pCL15-*pbp1*T		This study
pCL15-*pbp1T*-ANT		This study
pRB473-*recA lacZ*		This study
**Primers**		
recA-F2	CCTATTGACCCGGGATTAAAAGATG	This study
recA-R2	GTTGAAACCCCGGGACCTATAT	This study
recA-seq	GTTTGTTCGTTTTTGCGTTTTG	This study
lacZ-seq	CATTAAATGTGAGCGAGTAACAAC	This study
pbp1-term-F	TAAGTGTGGATCCAGAATTTTAAGGTAG	This study
pbp1-term-R	GGTGTACGAGCTCTCTTCATATG	This study
pbp1F-ans-XbaI	CATGGATCTAGAAAGAAGATTTAGAT	This study
pbp1R-ans-HindIII	CATGGAAAGCTTCCTAAATCTTGACC	This study
16S-F	TCCGGAATTATTGGGCGTAA	This study
16S-R	CCACTTTCCTCTTCTGCACTCA	This study
recA-F	GAGAAATCTTTCGGTAAAGGT	This study
recA-R	GTGAAGCGCTACTGTTGTCTTACC	This study
pbp1-GF	AGGTAGCGGTTTTGTGTCC	This study
pbp1-GR	TATCCTTGTCAGTTTTACTGTC	This study
pbp2-GF	TATTTAGCCGGTTTACCTCA	This study
pbp2-GR	TTTTGACGTTCTTCAGGAGT	This study
pbp3-GF	GTGGACCAACCTCATCTTTA	This study
pbp3-GR	CGGGAGACCCTTATTATTCT	This study
pbp4-GF	TGGTGCTAACTGCTTTGTAA	This study
pbp4-GF	GCTAAAGCTATCGGAATGAA	This study

Selection from heterotypic (HeR) to the homotypic (HoR) resistance phenotype from SA13011 and derivatives was performed as previously described [13], [15]. Briefly, the bacteria were grown in 5 ml of LB broth without antibiotic overnight. Cultures were then back-diluted to an optical density at 600 nm (OD_600_) of 0.05–0.1 in LB broth with or without sub-inhibitory concentration of β-lactam and non-β-lactam (Sigma-Aldrich) antibiotics, and grown at 37°C with shaking (180 rpm).

### Determination of mutation frequency

Mutation frequencies for resistance to rifampicin were determined during the selection process for SA13011-HeR and derivatives (−/+ OXA 0.5 µg/ml). Inoculated flasks were incubated at 37°C with shaking at 145 rpm; aliquots of 100 µl were taken at different time intervals, including 6, 27, and 33 h as we previously described [15], [21]. All of the variants were selected on TSA plates containing rifampicin 200 µg/ml and TSA plates using serial dilutions to determine CFU/ml. Mutation frequencies were expressed as the number of rifampicin-resistant mutants recovered as a fraction of the viable count. Three independent cultures were sampled in triplicate to minimize error caused by inter- and intra-sample variation.

### DNA manipulation and sequencing

Chromosomal DNA was extracted using the Qiagen genomic DNA preparation kit (Qiagen, Valencia, CA) according to the manufacturer's directions. Sequencing of all PCR amplification products and plasmids was performed by the Nucleic Acid Research Facility at GENEWIZ (Houston, TX). Sequence analysis of *recA* from SA13011 and derivative strains was performed using a set of primers previously described [22]–[24]. Consensus sequences were assembled from both orientations and DNASTAR Lasergene (Madison, WI). *S. aureus* N315 (accession # BA000018) was used as a positive control.

### Construction of *recA* promoter reporter activity assay

A promoter fragment of *recA* was amplified by employing recA-F2 and recA-R2 primers (Table 1). The PCR product, covering 398 bp upstream of the *recA* start codon (containing regulatory sequences of the gene, including LexA binding sites [25]) and 102 bp encoding for the first 34 amino acids of RecA, were ligated in front of the promoterless *lacZ* gene of pMC1871 [26]. The plasmid was verified by restriction enzyme digestion and PCR to determine the correct orientation, and transformed into competent *E. coli* TOP10 cells (Invitrogen Life Technologies, Carlsbad, CA). Colonies containing the *recA-lacZ* fusion were selected in tetracycline agar plates (10 µg/ml) and X-gal (5-bromo-4-chloro-3-indolyl-β-D-galactopyranoside; 40 µg/ml). The construct was digested with *PstI*, and ligated into the *PstI* restriction site of a shuttle vector, *pRB473*. The pRB473-*recA*prom-*lacZ* reporter was sequenced using *recA* seq and *lacZ* seq primers (Table 1) to verify the absence of mutations. The final construct was electroporated into *S. aureus* RN4220 and transduced into SA13011-HeR by 80α phage-mediated transduction [27]. The resulting strain harboring SA13011-HeR *recA*-lacZ was designated LMR-21 (Table 1).

### *pbp1* antisense sequence construct

To construct the antisense sequence of *pbp1*, a 336-bp fragment corresponding to the downstream region of *pbp1* and containing the transcriptional terminator of the gene, was amplified using Pbp1-term-F and Pbp1-term-R primers (Table 1). The PCR product was digested with *BamHI* and *SacI* and ligated into pCL15 [28]; the resulting construct was designated pCL15-*pbp1*T. A second fragment of *pbp1* covering 475-bp upstream and 146-bp downstream of the start codon was amplified using PBP1F-ans (containing the *XbaI* restriction site) and PBP1R-ans (containing the *HindIII* restriction site) primers. The PCR product was digested with *XbaI* and *HindIII* and cloned into the corresponding restriction sites of pCL15-*pbp1*T, yielding pCL15-*pbp1*T-ANT (Table 1). The pCL15-*pbp1*T-ANT construct was confirmed by restriction enzyme digestion and sequencing with PBP1R-ans and PBP1F-ans primers. The constructs pCL15-*pbp1*T-ANT and pCL15-*pbp1*T (empty-vector) were electroporated into *S. aureus* RN4220 and transduced into SA13011-HeR by 80α phage-mediated transduction. The resulting strains, SA13011-pCL15-*pbp1*T-ANT and SA13011-pCL15-*pbp1*T-EV (empty-vector), were designated LMR23 and LMR25, respectively.

### β-galactosidase assays

LMR-21 [SA13011-HeR(*recA*P::*lacZ*)] was grown as we previously described [15] during the process of HeR/HoR selection, in the absence and presence of sub-inhibitory concentrations (Table 2) of various β-lactam antibiotics, including OXA (0.5 µg/ml), CLOX (0.25 µg/ml), BAL (0.25 µg/ml), ZOX (32 µg/ml), FOX (2 µg/ml), and IMP (0.012 µg/ml); and non-β-lactam cell wall inhibitors including, BAC (32 µg/ml), DCS (8 µg/ml), and VAN (0.5 µg/ml). An equal number of bacteria were centrifuged, re-suspended in 750 µl of buffer Z (0.06M Na_2_HPO_4_, 0.04M NaH_2_PO_4_, 0.01M KCl, 1 mM MgSO_4_, 0.05M β-mercaptoethanol, pH 7.0), transferred into Lysing Matrix B tubes with silica spheres (MP Biomedicals, Santa Ana, CA), and disrupted using a FastPrep FP120 high-speed homogenizer (Thermo Fisher Scientific, Rockford, IL) for 40 sec at a speed setting of 6.0. Protein concentrations were determined using Protein Assay Reagent (Pierce, Thermo Fisher Scientific) per the manufacturer's recommendations.β-galactosidase activity was measured by using total protein extracted from cultures and assessed by 2-nitrophenyl β-D-galactopyranoside degradation (ONPG). As a positive control of the *recA* promoter activity, we used a protein sample extracted from LMR-21 grown in the presence of 0.005 µg/ml of mitomycin C, a well-known inducer of the SOS system [17], [29].

**Table 2:** Minimal inhibitory concentration (MIC) of SA13011-HeR *recA*-lacZ (LMR21) to antimicrobial agents used in the study.

ANTIBIOTICS	MIC value (µg/ml)
Oxacillin (OXA)	2
Cloxacilin (CLOX)	1
Ceftobiprole (BAL)	1
Ceftizoxime (ZOX)	128
Cefoxitin (FOX)	8
Aztreonam (ATM)	512
Cefotaxime (CTX)	8
Cefaclor (CEC)	8
Imipenem (IMP)	0.25
Bacitracin B (BAC)	128
D-cycloserine (DCS)	32
Vancomycin (VAN)	1

### Real-time Reverse Transcriptase -PCR

RNA extractions for real-time reverse transcriptase (RT)-PCR were performed as previously described [15], [30], [31]. Total RNA was extracted using a RNeasy Mini Kit (Qiagen, Valencia, CA); all RNA samples were analyzed by *A*_260_/*A*_280_ spectrophotometry and gel electrophoresis to estimate concentration and asses integrity, and cleaned of potential DNA contamination by treating them with DNAse per the manufacturer's recommendations (Ambion, Life Technologies, Austin, TX). Real time RT-PCR analysis was done using the SensiMix SYBR One-Step kit (Quantace/Bioline, Taunton, MA) according to the manufacturer's protocol. Gene expression was evaluated using *C*_T_ values converted to fold change and compared with a sample used as a reference (value = 1) using log_2_ −(ΔΔCt). The change (*n*-fold) in the transcript level was calculated using the following equations: Δ*C_T_* = *C_T(test DNA)_*−*C_T(reference cDNA)_*, ΔΔ*C_T_* = Δ*C_T(target gene)_*−Δ*C_T(16S rRNA)_*, and ratio  = 2^−ΔΔC^ *_T_* [32]. The quantity of cDNA for each experimental gene was normalized to the quantity of 16S cDNA in each sample as determined in a separate reaction. Each RNA sample was run in triplicate. Values represent the means of at least three biological replicates ± standard error of the mean (SEM), sampled in triplicate to minimize error by inter- and intra-samples. Differences between the mean values were analyzed using a one-way analysis of variance (ANOVA). A *P* value of <0.01 was considered statistically significant (*). Oligonucleotide primers are shown in Table 1.

### Measurement of DNA synthesis during HeR-HoR –β-lactam mediated selection

The rate of DNA synthesis in cells initiating growth was measured by following the incorporation of methyl-[3H]-thymine into cells uniformly labeled with the same precursor. Cultures of SA13011 cells were grown in the absence and presence of OXA (0.5 µg/ml) during the selection process. When SA13011-HoR reached OD_600 nm_: 0.13, cultures of SA13011-HeR were back-diluted and adjusted to the same OD with pre-warmed medium. Both SA13011-HeR/-HoR cultures were supplemented with the radioactive precursor (1 µCi/ml) at the same specific activity and concentration. Triplicate samples were removed at different time-intervals and collected for DNA extraction (Qiagen, Valencia, CA); eluted, labeled DNAs were transferred to a Whatman filter paper and, after drying, radioactivity was measured by liquid scintillation. An unlabeled set of inoculated flasks (SA13011-HeR ± OXA 0.5 µg/ml) were grown as controls to monitor growth and for measurement of CFU/ml at the same time intervals.

### Time kill analyses

Bactericidal synergy assays for CTX, BAL, FOX, and IMP were performed using MH broth with an initial inoculum of 1×10^6^ CFU/ml at ½ MICs (based on individual strain E-test data shown in Table 2), as previously described [33]. Aliquots of each culture were serially diluted and plated for CFU determination at time intervals 0, 2, 4, 6, 8, and 24 h. A minimum of two independent experiments were run for each combination.

### Wax worm infection

*Galleria mellonella* larvae possess an immune system with reasonable homology to vertebrates containing hemolymph analogous to blood, transports nutrients, hemocytes and immune molecules [34], [35]. These tissue types are similar to those encountered by *S. aureus* during invasive infections in humans. Groups of larvae of *G. mellonella* (10/group) were inoculated with 10 µl of the bacterial suspension of SA13011-HoR strain (1.5×10^6^ CFU) as previously described [34]. All larvae were confirmed to be alive at 2 h post-inoculation (here designated 0 h). Then, The first clinically-recommended treatment doses [36], [37] of IMP (10 mg/kg), NAF (5 mg/kg), CTX (10 mg/kg), and BAL (5 mg/kg), or the combinations IMP/NAF, IMP/CTX, and IMP/BAL, were administered in PBS into the right hind most proleg, and re-incubated for 24 h at 37°C. In addition, control groups included one group of larvae that had been inoculated with live bacteria that received PBS only as treatment, and the uninfected control group, which received PBS treatments to control for multiple injections. Repeat treatment doses were given at 24 and 48 h. Worms were checked daily, and deaths were recorded for a total of 10 days. A minimum of three independent experimental runs were performed for each IMP/β-lactam combination. The survival data were plotted using the Kaplan–Meier method.

## Results

### Activation of *recA*-mediated SOS induction during HeR/HoR selection

**Figure 1:**
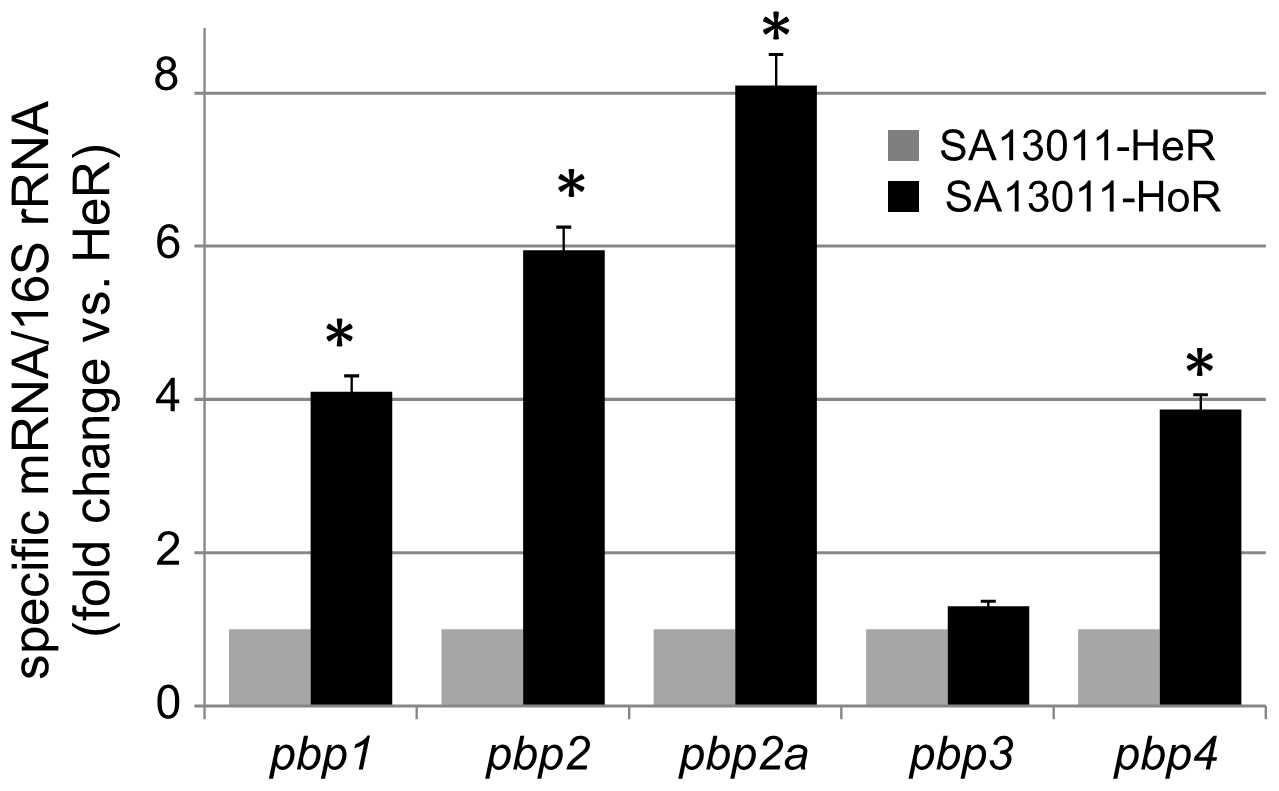
Quantitation of *pbp*1-4 and *pbp2a* mRNAs by real-time RT-PCR was performed by using the corresponding set of primers described in Table 1. RNAs were extracted from SA13011-HeR/HoR collected at the exponential phase of growth, as described in Materials and Methods. Relative fold change values representing the means of at least three biological replicates of specific mRNAs ± standard error of the mean (SEM), sampled in triplicate to minimize error by inter- and intra-samples, are shown on the vertical axis; 16S rRNA was used as an internal control. *: indicates significantly higher than SA13011-HeR, *P*<0.01.

In our previous studies, we determined that β-lactam-mediated activation of the SOS response during HeR/HoR selection involved increased expression of *recA* [15]. As mentioned above, the present study was designed to test the hypothesis that β-lactam-induced SOS response in heterogeneous MRSA may originate when the cell wall integrity is compromised. To determine the impact of β-lactam-mediated HeR/HoR selection on the expression of PBPs, we analyzed their expression profile by real-time RT-PCR analysis. RNA samples from SA13011-HeR/HoR cells were collected at the exponential phase of growth (OD_600_ = 0.6–0.7). As shown in Figure 1, selection of the HoR derivative resulted in increased expression of most of the *pbp* genes, including *pbp1, pbp2, pbp2a, and pbp4*; levels of *pbp3* remained almost undetectable under any condition and displayed no changes in expression. Thus, this analysis showed that OXA-mediated HeR/HoR selection is characterized by a generalized increase in the expression of most of the genes coding for PBPs.

To investigate β-lactam-mediated *recA* activation, we generated a SA13011-HeR derivative strain harboring a *recA-lacZ* reporter gene [SA13011-HeR(*recA*P::*lacZ*), LMR21]. The construct contains the upstream *recA* promoter followed by a portion of the 5′ *recA* coding region ligated to a *lacZ* gene as described in Materials and Methods. Phenotypic analysis of LMR21 is shown in Table 2. To establish the role that different β-lactam antibiotics may play in activating the *recA* promoter, *recA* promoter-mediated β-galactosidase activity was determined in LMR21 cells exposed to sub-lethal concentrations (1/4 MIC; Table 2) of different antibiotics harvested at the exponential phase of growth (OD: 0.6). We based our choice on their specific PBP/s target affinity, i.e. nonselective: OXA and CLOX (PBP1-PBP3); selective: CTX (targets PBP2); BAL (PBP2a), CEC (PBP3), and FOX (PBP4). The other antibiotics used were based on the fact that they are directed against different steps of cell wall synthesis, although no PBPs mediated, including VAN (binding to C-terminal of D-Ala- D-Ala residues of the externally oriented lipid-linked peptidoglycan precursor), BAC (binds to undecaprenyl pyrosphospate [UPP], the lipid carrier for translocation of cell envelope precursors from cytosol to the extracellular surface of the cytoplasmic membrane), and DCS (interferes with D-alanine incorporation into peptidoglycan). As shown in Table 3, increased β-galactosidase activity (approx. 2–3-fold change) was observed in the presence of sub-inhibitory concentrations of both OXA (0.5 µg/ml) and CLOX (0.25 µg/ml), both agents targeting PBP1 and PBP3. By contrast, no changes in β-galactosidase activity were observed in the presence of FOX (PBP4), ZOX (PBP2), or BAL (PBP2a). Similarly, no *recA* promoter induction was observed with other cell wall targeting antibiotics, including CEC, CTX, BAC, VAN, or DCS (Table 3). These results suggested that inhibition of PBP1 and/or PBP3 by OXA and CLOX may be involved in *recA* activation occurring during HeR/HoR selection. To determine the specific contribution of each of these PBPs, we used IMP, which targets PBP1 [38], and cefaclor, a β-lactam specific for PBP3 [39]. While exposure of LMR21 cells to IMP resulted in a 2.7-fold increase in β-galactosidase activity, no induction of *recA* promoter activity was observed in cells exposed to cefaclor (Table 3). Furthermore, and in agreement with a role of PBP1 on *recA* promoter activation, LMR21 cells exposed simultaneously to IMP and OXA (0.06 µg/ml and 0.5 µg/ml, respectively) displayed a 5.3-fold increase in *recA* promoter/β-galactosidase activity compared to a ∼2.5-fold increase produced by either IMP or OXA administered individually (Table 3). Together, these observations led us to postulate that PBP1, a common target of OXA and IMP [40], may play an important role in *recA*-mediated activation of the SOS system during β-lactam-mediated HeR/HoR selection.

**Table 3:** β-galactosidase activity (Miller units) of SA13011-HeR(*recA*P::*lacZ*) (LMR21) grown in the absence or presence of (1/4 MICs values) the corresponding antibiotics.

ANTIBIOTICS	Fold change (Miller units)
Oxacillin (OXA)	2.2±0.1
Cloxacillin (CLOX)	2.5±0.1
Cefoxitine (FOX)	0.8±0.05
Ceftizoxime (ZOX)	1.0±0.08
Cefaclor (CEC)	0.9±0.08
Cefotaxime (CTX)	0.1±0.003
Ceftobiprole (BAL)	0.7±0.04
Vancomycin (VAN)	0.9±0.06
Bacitracin A (BAC)	0.6±0.002
D-cycloserine (DCS)	0.9±0.07
Mitomycin C (+control)	4.8±0.2
Imipenem (IMP)	2.7±0.1
IMP+OXA	5.3±0.25

The number of Miller units is expressed in fold change. Values represent the mean ± SD of three independent replicates.

### PBP1 is required for β-lactam-mediated *recA* increased expression

**Figure 2:**
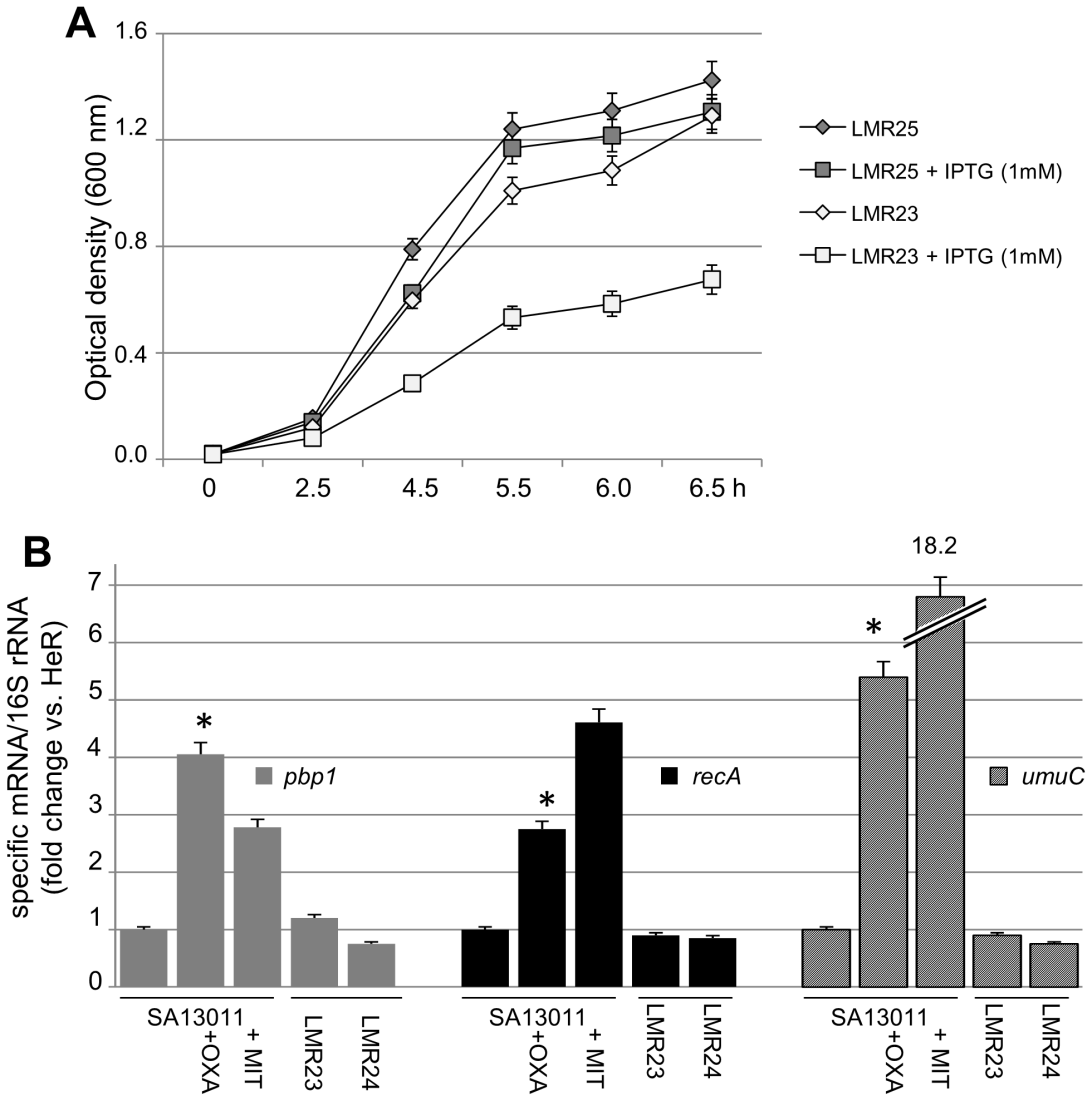
(A) Kinetic of growth of the LMR23 (*pbp1*-antisense) in the presence and absence of IPTG (1 mM). IPTG was added to the cultures and cells were collected at the indicated time intervals for OD measurement. LMR25 (empty-vector) was used as a control. (B) Quantitation of *pbp*1, *recA*, and *umuC* mRNAs by real-time RT-PCR. RNAs were extracted from SA13011-HeR −/+ OXA, and LMR23+IPTG, −/+ OXA (LMR24), collected at the exponential phase of growth, as described in Materials and Methods. Relative fold change values of specific mRNAs are shown on the vertical axis; 16S rRNA was used as an internal control. Values represent the means of at least three biological replicates ± standard error of the mean (SEM), sampled in triplicate to minimize error by inter- and intra-samples, respectively. *: indicates significantly higher than SA13011-HeR, *P*<0.01. Specific sets of primers are described in Table 1. MIT: mitomycin C (0.005 µg/ml), a well-known inducer of the SOS system and modulator of *recA* promoter, was used as a positive control.

The observations that OXA and IMP, by interfering with PBP1, were the two β-lactams increasing *recA* activity support the notion that, during β-lactam-induced HeR/HoR selection, PBP1 may have a critical role in the activation of the RecA-mediated SOS response. To test this hypothesis, functional assays where conducted by using an inducible PBP1-specific antisense RNA system. The choice of an IPTG-inducible PBP1 antisense construct to reduce *pbp1*expression was based on the fact that PBP1 is an essential enzyme and, as shown by Pereira et al. [7], its constitutive down-regulation may result in cell death [6], [7]. The resulting strains, SA13011-HeR+pCL15-*pbp1*T-ANT (LMR23) and the corresponding empty-vector control LMR25 (SA13011-HeR+pCL15-*pbp1*T), were first evaluated for their capacity to grow −/+ IPTG (1 mM) (Figure 2A). Both LMR23/25 strains were grown overnight in the absence of IPTG and then back-diluted to an OD_600 nm_: 0.020; after 2 h, IPTG (1 mM) was added to the cultures and cells were collected at the indicated time intervals for OD measurement. Interestingly, inducible expression of the *pbp1* antisense (LMR23+IPTG 1 mM) significantly impaired and delayed the growth of the strain as compared to the corresponding empty vector-expressing cells (LMR25 ± IPTG), or LMR23 growing in the absence of the inducer IPTG (Figure 2A).

The expression of *pbp1* was monitored by real-time RT-PCR. RNAs were extracted from cells grown −/+ IPTG (1 mM) in the absence or presence of OXA (0.5 µg/ml) and collected at the exponential phase during OXA-mediated HeR/HoR selection. As shown in Figure 2B, IPTG-induced *pbp1*-antisense expression abolished the OXA-induced increase in *pbp1* expression. Importantly, inactivation of the *pbp1* gene also abolished OXA-induced increased *recA* expression (Figure 2B).

In previous studies, we demonstrated that β-lactam-mediated triggering of the SOS response was associated with an increase in the mutation rates [15]. To determine whether inactivation of PBP1, which blocked OXA-induced *recA* expression, may affect the β-lactam-mediated change in mutation rate, the number of mutants in strains LMR23 and corresponding controls [SA13011-HeR and LMR25 (pCL15-T-empty-vector)], were measured. Frequency of mutation was determined by growing the strains in the absence and presence of sub-inhibitory concentrations of OXA (0.5 µg/ml) at 6-, 27-, and 33-h time intervals during the HeR/HoR selection process. We used the events of occurrence of rifampicin-resistant mutants as a marker for mutation frequency expressed as the ratio of rifampicin-resistant mutants recovered as a fraction of the viable count (Table 4) [15], [21], [41], [42]. While exposure to sub-inhibitory concentrations of OXA determined a ∼4-log increase in mutation rates in both SA13011-HoR and LMR26 (LMR25-empty vector+OXA) control strains at the 33 h interval (i.e., 1.4×10^−9^/2.8×10^−5^, 1.4×10^−9^/2.3×10^−5^, SA13011-HeR/HoR, LMR25/26, respectively), these changes were suppressed in cells expressing the inducible *pbp1*-antisense (i.e., 1.8×10^−9^/2.5×10^−8^, LMR23/LMR24+IPTG/OXA, respectively) (Table 4).

**Table 4:** Mutation rate analysis during OXA-induced HeR/HoR selection of parents and homogenous derivatives of SA13011, LMR25, and LMR23 strains.

	6 h	27 h	33 h
SA13011-HeR	8.7×10^−8^	2.6×10^−9^	1.4×10^−9^
SA13011-HeR+OXA * (HoR)	4.0×10^−7^	1.6×10^−5^	2.8×10^−5^
LMR25+IPTG	3.4×10^−8^	1.6×10^−9^	1.4×10^−9^
LMR26 (LMR25+IPTG+OXA*)	1.7×10^−7^	1.0×10^−5^	2.3×10^−5^
LMR23+IPTG	2.5×10^−8^	2.1×10^−9^	1.8×10^−9^
LMR24 (LMR23+OXA)	8.7×10^−7^	2.1×10^−5^	8.7×10^−5^
LMR24 (LMR23+OXA)+IPTG	2.3×10^−8^	5.6×10^−8^	2.5×10^−8^

Mutation frequency was determined using rifampicin selection and expressed as a ratio of rifampicin resistance as a fraction of viable cells, during time points at 6, 27, and 33 h of the selection process, −/+OXA 0.5 µg/ml (*).

Within the SOS-mediated response, the UmuC error-prone polymerase has been identified in *S. aureus* as one of the enzymes involved in the increased mutation rate observed after SOS induction by ciprofloxacin [17]. The expression levels of *umuC* were determined in the same set of samples described above. As shown in Figure 2B, and consistent with the mutation rates shown in Table 4, expression of *umuC*, which underwent a significant increase in SA13011+OXA, remained unchanged in the absence of *pbp1* induction (i.e. LMR23 +OXA/IPTG, Figure 2B). Together, these results highlight the central role played by PBP1 in β-lactam-mediated RecA activation and subsequent triggering of the SOS response and increased mutation rate.

### Effect of oxacillin in DNA synthesis during β-lactam-mediated HeR/HoR selection

**Figure 3:**
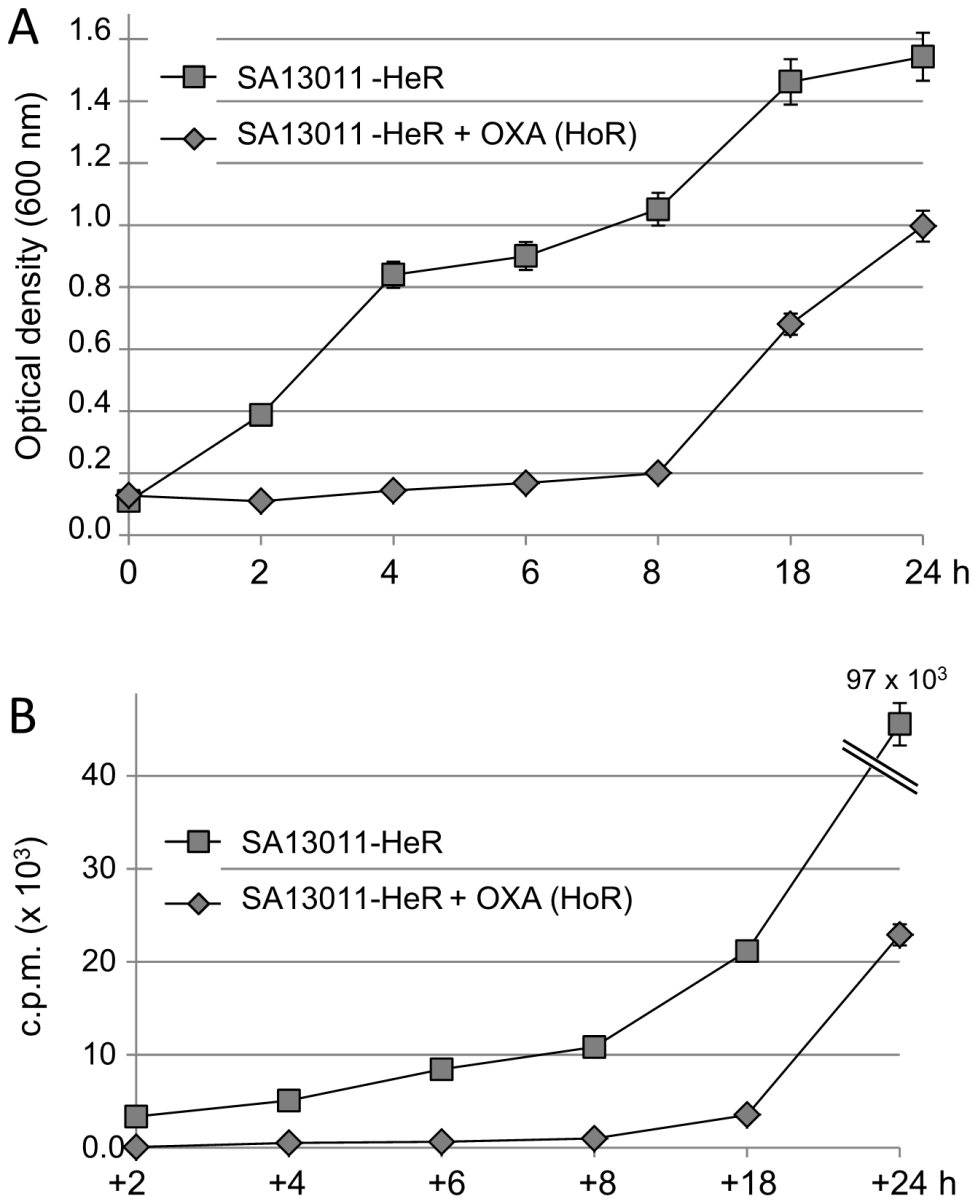
Time-course analysis of growth (A) and rate of DNA replication (B) was performed in SA13011-HeR −/+ OXA (0.5 µg/ml) during β-lactam-induced HeR/HoR selection. Bacteria were grown −/+ OXA (0.5 µg/ml) overnight. Cultures without OXA (HeR) were then diluted to an optical density at 600 nm (OD_600_) equivalent to the culture growing with OXA (HoR) and continued to grow at 37°C with shaking. (B) Both SA13011-HeR/-HoR cultures were supplemented with the radioactive methyl-[3H]-thymine precursor (1 µCi/ml); triplicates samples were removed at different time-intervals and collected for DNA extraction, eluted and transferred to Whatman paper filters; after drying, radioactivity was measured by liquid scintillation. Values represent the means of three biological replicates ± standard error of the mean (SEM), sampled in triplicate to minimize error by inter- and intra-samples, respectively.

There is evidence that PBP1 is a major player involved in cell division and separation in *S. aureus* [6], [7]. Furthermore, in *E.coli*, studies have shown that DNA replication and cell division are tightly coupled and synchronized processes [43], [44]. Taking into account the observations showing a marked delay in growth provoked by the inducible reduction of *pbp1* gene expression (Figure 2A), we investigated the status of DNA replication in SA13011 undergoing OXA-mediated selection. DNA synthesis was measured by following the incorporation of methyl-[3H]-thymine into SA13011 cells grown in the absence and presence of OXA (0.5 µg/ml) during the selection process, as described in Methods. Consistent with previous studies investigating the phenomenon of OXA-mediated HeR/HoR selection [21], the selected homotypic derivatives displayed slow kinetic of growth when compared to the heterotypic variant (e.g., OD_600 nm_ 8 h of culture: 1.051/0.2; CFU/ml: 7.5×10^8^/7.2×10^5^, SA13011-HeR/-HoR, respectively; Figure 3A). OXA-mediated effects on cell growth were also reflected in the rate of DNA synthesis as shown by a significant reduction in the rate of methyl-[3H]-thymine incorporation into DNA in cells growing in the presence of OXA [e.g., cpm (×10^3^) 8 h: 10.9/0.9, SA13011-HeR/-HoR, respectively; Figure 3B].

### Antibacterial efficacy of combining PBP1 and PBP2/PBP2a inhibitors against HoR-MRSA strains

**Figure 4:**
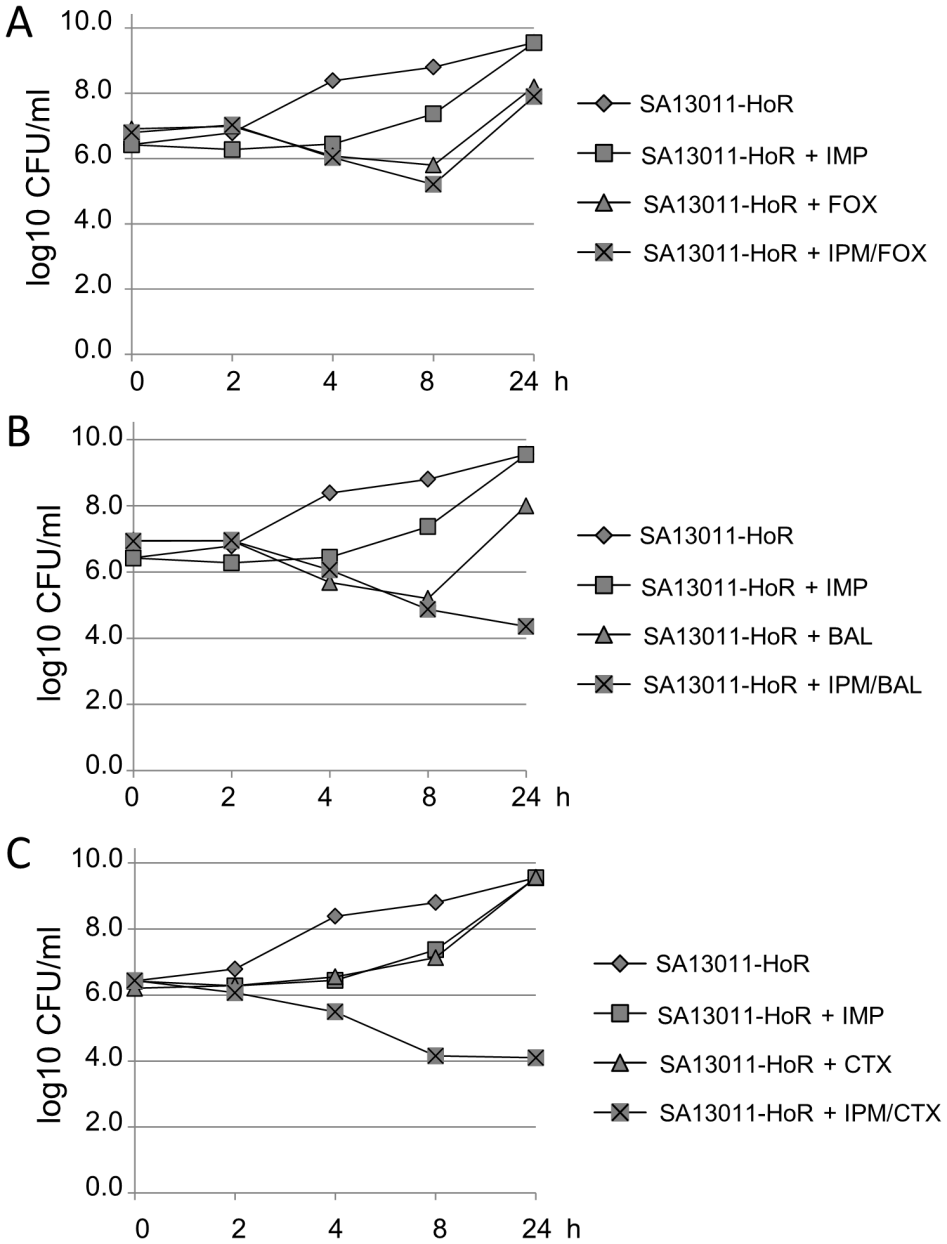
Analysis of antibacterial efficacy against SA13011-HoR. Synergy time kill analysis was performed using Mueller-Hinton (MH) broth with 10^6^ CFU/ml inoculums at 0, 2, 4, 6, 8, and 24 h. Antibiotics were used as specified at the following concentrations: IMP: 0.12 µg/ml; FOX: 4 µg/ml; BAL: 0.5 µg/ml, and CTX: 4 µg/ml. A minimum of three independent experimental runs were performed for each IMP/β-lactam combination.

The results showing that PBP1 plays a pivotal role in determining the signaling responsible of increasing the mutation rate, a critical step in β-lactam-mediated HeR/HoR selection, plus the fact that this process is accompanied by a compensatory increase in the expression of not only *pbp1* but also *pbp2*, *mecA*, and *pbp4* (Figure 1), led us to hypothesize that simultaneous inactivation of PBP1 (because of its effects on the *recA*/SOS response) and one of the other PBPs (e.g., PBP2, MecA, or PBP4) may result in synergistic antibacterial effects and provide an effective therapy against HoR-MRSA strains. To test this concept, we used both *in-vitro* synergy time-kill curves and an *in-vivo* wax worm model, as previously described [34], [45]. *In-vitro* synergy-kill analysis of interactions between IMP and β-lactams currently used in clinical therapeutics (CTX [PBP2], BAL [PBP2a], and FOX [PBP4]) against SA13011-HoR strain were performed at 0, 2, 4, 8, and 24 h using MH broth; an initial inoculum of 1×10^6^ CFU/ml in the presence of ½ MICs (Table 1) of IMP and each of the β-lactams were tested. The size of the inoculum was determined by matching bacterial counts commonly achieved in all target tissues of animals with experimental infective endocarditis [46], [47]. At these concentrations, neither antibiotic alone displayed significant bactericidal effects (Figure 4A–C). While IMP and FOX administered together have no bactericidal effect (Figure 3A), the combination of IMP with either BAL (PBP2a) or CTX (PBP2) were highly synergistic as demonstrated by cell killing at 24 h ≥4-log CFU *vs.* single agents and the initial inoculum (Figure 4B–C). The population remaining after exposure to either IMP/BAL or IMP/CTX does not represent a subpopulation of resistant cells and correspond to metabolic inert cells, as we verified by E-Test (data not shown). These results suggested that the combination between IMP (PBP1) and antibiotics blocking either PBP2 or PBP2a may have a major impact as an anti-infective alternative in HoR-MRSA strains.

**Figure 5:**
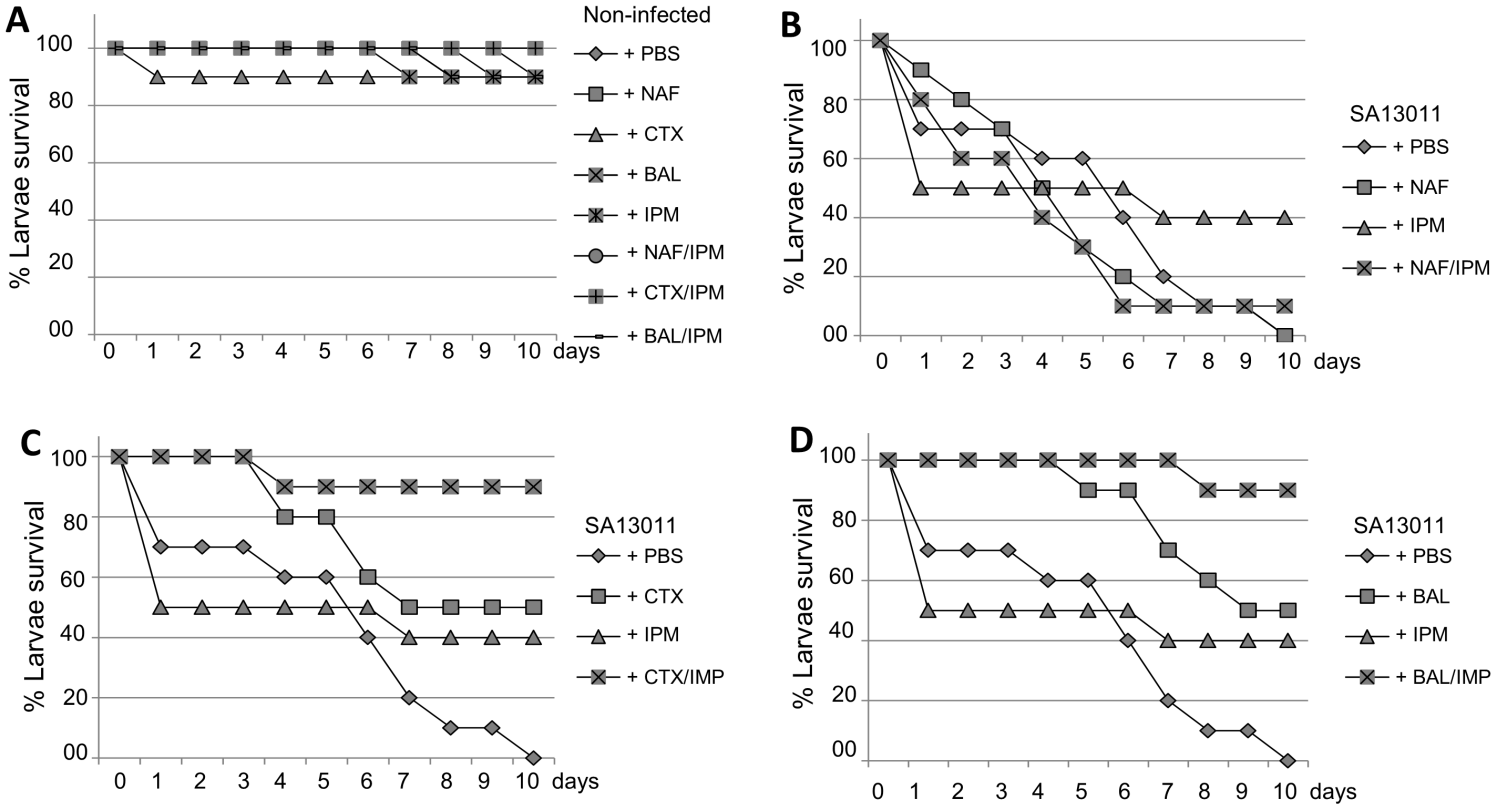
Treatment effect using *Galleria mellonella*. Groups of larvae (10/group) were inoculated with either 10 µl of PBS (uninfected control group; A) or bacterial suspension containing 1.5×10^6^ CFU/ml of 13011-HoR (B–D) into the last proleg and incubated for 2 h at 37C. After this, 10 µl of IMP (10 mg/kg), NAF (5 mg/kg; B), CTX (10 mg/kg; C), BAL (5 mg/kg; D), or the corresponding IMP/β-lactam were administered (time 0) into the right hind-most proleg, and re-incubated for 24 h at 37°C. PBS treatment was given as a control with multiple injections. The treatment was repeated after the first 24 h incubation. Worms were checked daily and recorded for any deaths for a total of 10 days. A minimum of three independent experimental runs were performed for each experiment. Survival data were plotted using the Kaplan-Meir method.

To investigate whether regimens identified by *in-vitro* time-kill analysis combining IMP with β-lactams targeting either PBP2a (BAL) or PBP2 (CTX) may enhance the *in-vivo* therapeutic efficacy over that of each single agent against HoR-MRSA strains, we used a model of wax worms [34], [45], [48]. Larvae of the greater wax moth (*Galleria mellonella*) were shown recently to represent an alternative to vertebrates as a host model for studying pathogenic microbes, virulence, and therapeutic regimens [45], [48], [49], including the efficacy of antistaphylococcal agents [34]. Groups of larvae (10/group) were inoculated with a bacterial suspension containing SA13011-HoR (10^5^ bacteria/worm) as we previously described [48], incubated for 2 h at 37°C, after which IMP (10 mg/kg), BAL (5 mg/kg), CTX (10 mg/kg), or the corresponding combinations IMP/BAL, and IMP/CTX were administrated (0 h), and reincubated for 24 h at 37°C. Additionally, a group was treated with NAF (5 mg/kg; targets both PBP1 and PBP3; OXA or CLOX were replaced by NAF because of its clinical use recommendation) and IMP/NAF. An uninfected control group received PBS treatment to control for multiple injections. After the first 24 h incubation, treatment was repeated. Worms were checked daily, and recorded for any deaths for a total of 10 days. Uninfected worms treated with PBS, single drugs or the combinations IMP/NAF, IMP/CTX, or IMP/BAL showed 100/90% survival at day 10 of treatment (Figure 5A). On the other hand, groups of HoR-MRSA-injected worms untreated (PBS), or treated with single drugs, displayed low survival rates (≤50–10%, day 10; Figure 5B). By contrast, groups of worms treated with the combinations IMP/CTX and IMP/BAL resulted in survival rates of 90% at day 10 (Figure 5B). Interestingly, the combination of IMP/NAF, in which both antibiotics target PBP1 (PBP3 expression levels were very low and not affected in the SA13011-HoR strain; Figure 1) was ineffective. Thus, these results were in agreement with data from the *in-vitro* time-kill experiments (Figure 4) showing significant synergistic interactions between IMP, which targets PBP1, and β-lactams targeting PBPs whose expression levels, as a compensatory mechanism, appeared increased, i.e. PBP2 (CTX) or PBP2a (BAL).

## Discussion

Resistance in *S. aureus* to widely used antibiotics can be achieved through the selection of single mutations or through gene acquisition by horizontal transfer [17], [50]. Both of these processes have been linked to the RecA-mediated SOS response [17], [50]. The bacterial SOS response is a conserved regulatory network that is induced in response to DNA damage [17]. Its activation *in vitro* results in the emergence of resistance to antibiotics, an observation that has led to the speculation that this is an important clinical mechanism of resistance acquisition [51]. In previous studies, we demonstrated that, in clinical heterogeneous MRSA strains, the SOS response is activated in response to exposure toβ-lactam antibiotics, a mechanism associated with the selection of highly resistant derivative variants [15]. Taking into account that β-lactams are not DNA damage agents but cell wall inhibitors, we hypothesized that inhibition of the cell wall machinery may be involved in the triggering of SOS-mediated *recA* activation. The present results showing that *recA* was activated in cultures treated only with antibiotics targeting PBP1 (i.e., IMP or OXA), but not other PBPs (i.e., PBP2, -2a, -3, or 4), suggest that interference with this particular PBP plays a key role in β-lactam-mediated triggering of the SOS response. These results are consistent with evidence showing the role that PBP1 plays in cell division and separation in *S. aureus* [6], [7], highlighting the extent to which targeting it with β-lactams affects the growth of cells under antibiotic pressure. In the context of PBP1 inactivation by exposure to β-lactams, studies in *E.coli* have shown the synchronization existing between DNA replication and cell division [43], [44]. As mentioned above, despite that β-lactams are not considered DNA damaging agents, and as our results suggest, and it may be plausible to postulate that by interfering with PBP1 functions, β-lactam antibiotics may lead to cell division arrest and, in turn, could affect replication and generate the presence of single-stranded DNA, which may then contribute to the signals involved in the triggering of the SOS response and hypermutable state. Further studies are needed to test this hypothesis and are currently planned in our laboratory.

In the present work, we demonstrated that inhibition of PBP1 was linked to both *recA* activation and the concomitant increase in mutation rate, a critical step in β-lactam-mediated selection of the highly resistant HoR phenotype. Moreover, following PBP1 inhibition and *recA* activation, compensatory up-regulation of *pbp2*, *pbp2a*, and *pbp4* were observed. This observation raised the question of whether simultaneous targeting of PBP1 and compensatory PBP2, PBP2a, or PBP4 may provide a therapeutic advantage to limit the growth of HoR-MRSA, both *in-vitro* and *in vivo*. Our results indicate that, in fact, combining IMP (PBP1) with either CTX (PBP2) or BAL (PBP2a) results in a synergistic activity against HoR-MRSA. By contrast, simultaneous inhibition of PBP1 (IMP) and PBP4 (FOX), or using a combination of IMP/NAF, where both compounds target PBP1, did not result in increased antibiotic activity. Together, these observations were consistent with the notion that PBP1, which determines the signaling that leads to RecA activation and increased mutation rate, requires the cooperation of both PBP2 and MecA in order to sustain the highly resistant HoR-MRSA phenotype. Furthermore, this rationale is consistent with the idea that PBP2A is not the sole determinant for β-lactam resistance in MRSA, as previously suggested [11], [12], [52]. In addition, these results also show that, although PBP4 has been shown to be essential for expression of resistance in CA-MRSA, it does not play a significant role in heterogeneous MRSA strains which are mostly linked to HA-MRSA infections [14].

It is well known that β-lactam antibiotics are inducers of the SOS response; [53], [54] however, less is known about the relationship between SOS response and cell wall-mediated response in MRSA strains. The present work provides evidence that β-lactam-induced impairment of PBP1 function is responsible for generating the signal and triggering SOS response-mediated RecA activation, which, in turn, leads to an increase in the mutation rate. In summary, the present results offer new insights into the complex biology underlying β-lactam-induced HeR/HoR selection and acquisition of the highly resistant MRSA phenotype.
